# Cardiac Optogenetics: Enhancement by All-*trans*-Retinal

**DOI:** 10.1038/srep16542

**Published:** 2015-11-16

**Authors:** Jinzhu Yu, Kay Chen, Rachel V. Lucero, Christina M. Ambrosi, Emilia Entcheva

**Affiliations:** 1 Department of Biomedical Engineering, Stony Brook University, Stony Brook, NY.

## Abstract

All-*trans*-Retinal (ATR) is a photosensitizer, serving as the chromophore for depolarizing and hyperpolarizing light-sensitive ion channels and pumps (opsins), recently employed as fast optical actuators. In mammalian optogenetic applications (in brain and heart), endogenous ATR availability is not considered a limiting factor, yet it is unclear how ATR modulation may affect the response to optical stimulation. We hypothesized that exogenous ATR may improve light responsiveness of cardiac cells modified by Channelrhodopsin2 (ChR2), hence lowering the optical pacing energy. In virally-transduced (Ad-ChR2(H134R)-eYFP) light-sensitive cardiac syncytium *in vitro*, ATR supplements ≤2 μM improved cardiomyocyte viability and augmented ChR2 membrane expression several-fold, while >4 μM was toxic. Employing integrated optical actuation (470 nm) and optical mapping, we found that 1–2 μM ATR dramatically reduced optical pacing energy (over 30 times) to several μW/mm^2^, lowest values reported to date, but also caused action potential prolongation, minor changes in calcium transients and no change in conduction. Theoretical analysis helped explain ATR-caused reduction of optical excitation threshold in cardiomyocytes. We conclude that cardiomyocytes operate at non-saturating retinal levels, and carefully-dosed exogenous ATR can enhance the performance of ChR2 in cardiac cells and yield energy benefits over orders of magnitude for optogenetic stimulation.

All-*trans*-Retinal (ATR) is produced as part of the retinoid metabolism in mammalian cells, starting with exogenous sources of Vitamin A^1^. Retinoids, and retinoic acid (RA), in particular, are powerful regulators of cell function. ATR also serves as the light-sensing element (the chromophore) for all opsins, including mammalian and microbial ones. Depolarizing and hyperpolarizing light-sensitive ion channels and pumps of microbial origin have become the basis of the recently emerging field of “optogenetics”^2^,^3^,^4^,^5^,^6^,^7^ and have direct relevance to experimental manipulation of cardiac electromechanics^5^,^6^,^8^,^9^,^10^,^11^,^12^,^13^,^14^,^15^,^16^,^17^,^18^ and molecular signaling in the heart^19^ by light with high spatiotemporal precision and cell specificity.

ATR availability is necessary and essential for the proper function of Channelrhodopsin-2 (ChR2)^20^ and its gain of function variant ChR2(H134R)^21^, the most commonly used opsin. More specifically, ATR serves as a co-factor in the formation of functional ChR2 channels, covalently binding the pore-forming protein complex, the channelopsin. Upon photon absorption, ATR isomerizes to 13-*cis*-retinal and facilitates the opening of the channel. In current biophysical models of ChR2 and related mutants^14^,^22^, it is assumed that ATR is not a limiting step in the kinetics and the overall operation of the ion channel, as the retinal isomerization process is near-instantaneous^23^. Instead, ATR availability can be viewed, simplistically, as a scaler of ChR2 current (I_ChR2_) magnitude by virtue of increasing the average number of functional channels in the cell membrane at any given time, and therefore increasing the maximum macroscopic conductance for ChR2. Considering such purported scaling of I_ChR2_ by ATR availability, it is unclear if supplemental ATR can result in a corresponding improvement of optical excitability when applied to cells and tissues.

Nagel and colleagues found that certain experimental model organisms, e.g. C. elegans^21^ and D. melanogaster^24^, lack endogenous ATR that is capable of forming complexes with opsins, and therefore opsin functionality was only possible with ATR supplementation via the diet. However, in most mammalian cells and tissues *in vitro* and *in vivo*, availability of retinal has not been viewed as a limiting factor and optical excitation has been possible without ATR supplementation^6^ or with other supplements that included vitamin A^25^. Nevertheless, considering that ATR in nonretinal and nonembryonic tissues is only present in small amounts (<0.5 nmol/g)^26^, it is feasible to expect that exogenous ATR may modulate the optogenetic response, at least in some tissues, such as cardiac muscle, which was found to contain lower amounts of endogenous retinoids compared to liver, kidney, adipose and brain tissue, based on tissue measurements with chromatography and UV techniques (HPLC/UV)^26^,^27^. Given ATR’s diverse roles in different tissues, it is important to investigate its specific effects on cardiac electrophysiology and the possibility of its use as a potent light sensitizer in applications of optogenetics to the heart.

Therefore, in this study, we set out to explore the *in vitro* use of exogenous ATR as a photosensitizer in cardiomyocytes (CMs), genetically modified to express ChR2, and its potential to substantially reduce optical stimulation energy.

## Results

### Cardiomyocyte viability upon ChR2 expression and ATR treatment

We first confirmed that the genetic modifications of cardiomyocytes with ChR2 (Ad-ChR2-eYFP virus and its infection process) were benign. Using optimized multiplicity of infection^11^, ChR2-CM with no exogenous ATR had the same high viability rate as CM control, i.e. about 11% death as indicated by PI fluorescence image analysis.

**Figure 1:**
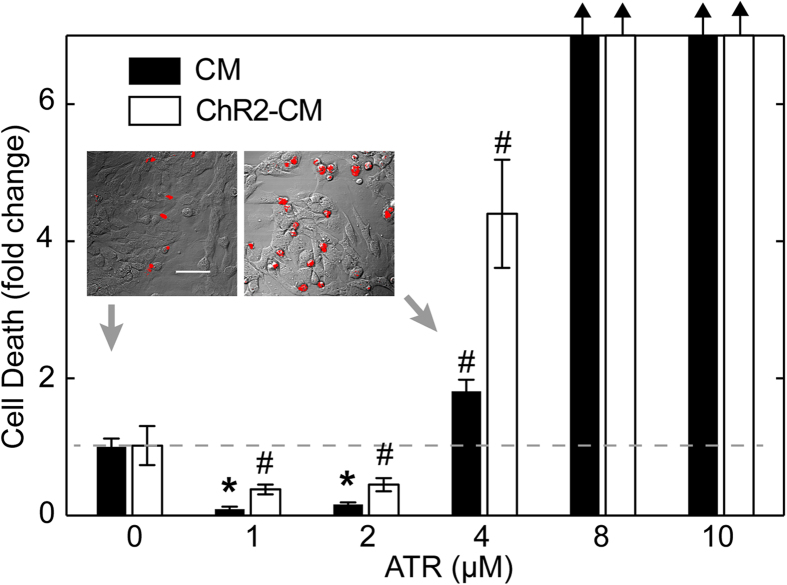
ATR effects on cardiomyocyte viability. Cell death assessed by quantifying fluorescence of PI; staining was done on the 4^th^ day after isolation and infection (n = 8–10 per group). Inset shows two examples of PI-stained dead cell (red) in control CM without ATR (0 μM) and control CM with 4 μM ATR overlaid onto DIC images. Scale bar is 50 μm. Under typical culturing conditions, CM without ATR (0 μM) exhibited 11.3% dead cells, and served as the reference of fold change (grey dash line) for other conditions with and without supplemental ATR. Data are presented at mean ± SEM. Black arrows indicate large fold change due to no cell survival above 4μM. (*) denotes significant difference at p ≪ 0.001, and (#) indicates significant difference at p < 0.01, where each group is compared to the control CM at 0 ATR.

Initial exploration of dosing with exogenous ATR revealed a narrow window of viable concentrations (Fig. 1). ATR supplementation greater than 4 μM was toxic to the cardiomyocytes, regardless of whether they expressed ChR2 or not, and caused complete cell death above 8 μM. However, ATR supplements less than 4 μM appeared to paradoxically improve cell viability even though control samples were quite healthy (Fig. 1): CM with 1 and 2 μM ATR supplements had only 0.1 × and 0.15 × the dead cell amount of CM control (virtually eliminating dead cells, i.e. <2% cell death), p ≪ 0.001 for both, and ChR2-CM with 1 and 2 μM ATR supplements had only 0.38 × and 0.45 × the dead cell amount of CM control, p < 0.01 for both. Intermediate ATR concentration of 4 μM increased cell death compared to cells without ATR supplementation, significant in both CM (1.8 × increase) and ChR-CM cells (4.4 × increase), p < 0.01 for both. Furthermore, it is worth noting that when ATR was present, the ChR2-CM samples had consistently lower viability than the CM samples at the same ATR, even though this was not the case for non-supplemented samples.

### ChR2 expression with ATR supplementation

**Figure 2:**
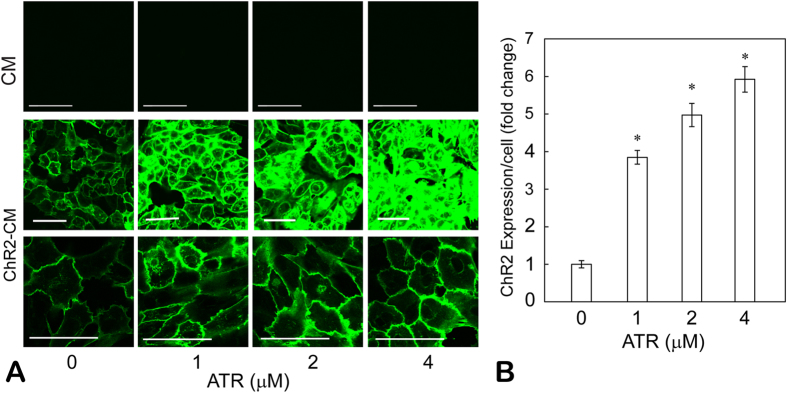
ATR effects on ChR2 expression in cardiomyocytes. (**A**) Fluorescent images of control CM and ChR2-CM, supplemented with viable concentrations of ATR (1–4 μM). ChR2 expression was confirmed by eYFP reporter fluorescence. Increasing ATR supplementation did not produce significant autofluorescence background (top row). Two gains, high (middle row) and low (bottom row), were selected during image acquisition to obtain the best dynamic range; these were maintained constant across ATR groups. Scale bars are 50 μm. (**B**) Quantification of ChR2 expression was done by green fluorescence (normalized per cell) within each field of view. Mean ± SEM of each condition were then normalized to the ChR2-CM without ATR group (n = 36 per group). (*) denotes significant difference from the control (zero ATR) and all other groups, p < 0.05.

Since ChR2 stabilization/degradation had been reported to be dependent on ATR availability^28^, we examined ChR2 expression efficiency (by eYFP reporter) in the viable range of ATR supplements, i.e. 0–4 μM. Employing previously reported infection methods^11^ without the addition of exogenous ATR, stable and robust transgene expression (>98%) was achieved within 48 hours^11^,^5^ (Fig. 2A, middle row, first image). We confirmed that in confocal image acquisition of fluorescence (eYFP), greater ATR concentrations did not result in an increase in autofluorescence (Fig. 2A, top row). ATR supplementation in ChR2-CM samples led to significant enhancement of ChR2 expression, indicated by the saturation of eYFP fluorescence at the higher doses, despite our attempt to select a gain that maximizes the dynamic range and accommodates all ATR dosing groups (Fig. 2A, middle row). Higher resolution images and lowered gain to optimize imaging of the ChR2-CM samples with 4 μM are also shown (Fig. 2A bottom row). Image analysis of eYFP-positive pixels (normalized per cell) showed 3.8 ×, 5 × and 5.9 × increase in ChR2 expression by the addition of 1, 2, and 4 μM ATR compared to ChR2-CM without retinal, p ≪ 0.001 in all cases (Fig. 2B). The eYFP fluorescence indicates the total number of expressed channels, including non-functional channelopsins without ATR, therefore the increases shown in Fig. 2 represent an upper bound for functional ChR2 channels. We assume that the data indicate a proportionally increasing number of stabilized functional ChR2 channels (fraction of the total reported channels), though increase in actual ChR2 current may be more modest.

### Augmentation of optical excitability of cardiac syncytia with ATR supplementation

**Figure 3:**
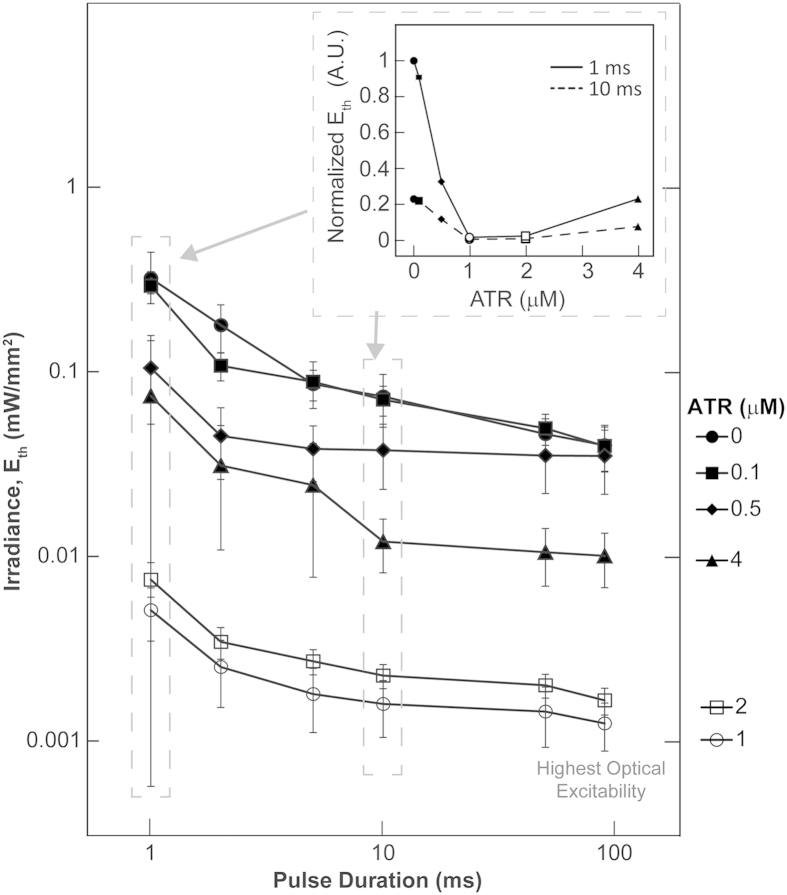
ATR modulation of optical excitability of ChR2-CM. Strength-duration curves (presented on a log-log scale) for ChR2-CM (at different ATR concentrations), linking the minimal irradiance, *E*_*th*_, needed to optically trigger propagating waves in cardiac syncytia at different light pulse durations. Data from n = 3 to 13 samples per data point, presented as mean ± SEM. The inset plot (non-log scale) illustrates the non-monotonic dependence of E_th_ on exogenous ATR concentration using data from 1 and 10 ms pulses. Highest optical excitability in ChR2-CM, or lowest *E*_*th*_, was achieved with 1 or 2 μM ATR. Two way ANOVA analysis (independent factors are pulse duration and ATR concentrations), setting α level at 0.05, produced 36 interaction comparisons (see Supplementary. Table S1).

To assess in detail the effects of ATR supplementation on modulating optical excitability, we conducted functional analysis in a total of 12 experimental groups, using six ATR doses in CM and ChR2-CM: 0, 0.1, 0.5, 1, 2, and 4 μM ATR. We quantified the minimal optical power (irradiance in mW/mm^2^) needed to elicit global excitation in cardiac syncytia *in vitro* at different light pulse durations (1 to 90 ms) by constructing strength-duration curves for optical excitation for each of the six ChR2-CM groups (Fig. 3). Irradiance thresholds were determined for successful capture of at least 10 consecutive beats by optical pacing.

The ChR2-CM group without retinal supplementation showed excellent excitability (less than 0.5 mW/mm^2^ for all pulse durations), on par with previous values for cell delivery to drive cardiac syncytia by light^15^. Gradual monotonic drop in *E*_*th*_ (increase in optical excitability) across pulse durations was observed as the ChR2-CM samples were dosed with 0.1, 0.5 and 1 μM of ATR, achieving a reduction in E_th_ of over 30 times for 1 μM ATR. Interestingly, increasing ATR concentration to 4 μM did not further reduce the threshold but reversed the trend towards the levels seen in un-supplemented ChR2-CM, despite increased ChR2 expression as seen in Fig. 2. This non-monotonic effect of ATR on optical excitability (see inset in Fig. 3) was in line with our findings on cell viability in Fig. 1. Detailed statistical comparison for all studied cases can be seen in Supplementary Table S1. In an alternative view, the optical strength-duration curves were further fitted to extract rheobase (the asymptotic irradiance at long pulse durations, i.e. minimal irradiance to stimulate) and chronaxie (a parameter related to the response kinetics of the biological system), Supplementary. Fig. S1. While rheobase followed the same trend of significant reduction in the 1 and 2 μM ATR groups, chronaxie did not reveal a distinct pattern.

### Functional electrophysiology effects of ChR2 expression and ATR supplementation in cardiomyocytes

**Figure 4:**
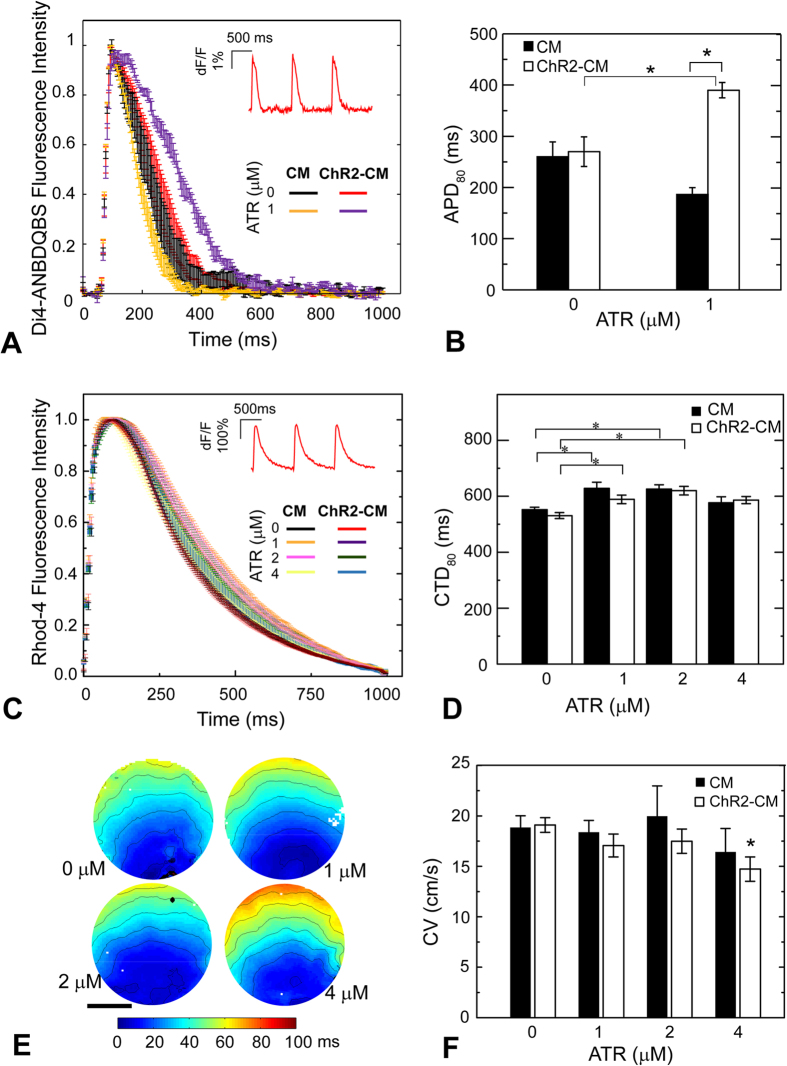
ATR effects on cardiomyocyte electrophysiology. (**A**) Action potentials in response to electrical pacing were imaged optically (using di4-ANBDQBS) and presented as mean ± SEM at each point for each group. (**B**) Quantified APD80 for control CM and ChR2-CM without ATR and with 1 μM ATR (highest optical excitability). Both A and B had n = 3–4 samples per experimental group. (**C**) Calcium transients in response to electrical pacing were imaged optically (using Rhod4-AM) and presented as mean ± SEM at each point for each group. (**D**) Quantified CTD80 for control CM and ChR2-CM at different ATR supplements. Both C and D had n = 7–33 samples for each of the eight experimental groups. (**E**) Example activation maps of ChR2-CM, following point electrical stimulation at the bottom; isochrones are 10 ms apart. Scale bar is 5 mm. (**F**) Quantified conduction velocity from the activation maps (n = 7–14 per group). (*) indicates significant difference at p < 0.05 compared to the respective control (zero ATR) or as indicated by the brackets.

We further examined the effects of ATR supplementation on CM electrophysiology by quantifying action potentials, calcium transients, and conduction properties. We compared optically-measured (via Di-4-ANBDQBS) action potential durations (APDs) of ChR2-CM to CM with the same treatment, Fig. 4A. At zero ATR, CM and ChR2-CM showed overlapping AP and similar APD80 of 260 ms and 270 ms, respectively; reassuring of the benign nature of the opsin expression and optogenetic activation. Addition of 1 μM ATR significantly prolonged the APD80 in ChR2-CMs by 44% (p < 0.05) compared to ChR2-CM without ATR (Fig. 4B). Similarly, at zero ATR, no significant differences were seen in calcium transient morphology between the CM and ChR2-CM groups across the tested conditions (Fig. 4C); CTD80 of CM and ChR2-CM were 552 ms and 530 ms, respectively (Fig. 4D). Supplementation with ATR of 1, 2, and 4 μM in control CM led to: 13.8% (p < 0.05), 13.3% (p < 0.05), and 4% (n.s.) prolongation of CTD_80_, respectively. In ChR2-CM samples, exogenous ATR supplementation also led to small but significant prolongation of CTD_80_ by approximately 10.9%, 11.6%, and non-significant 10% for the same tested concentrations (Fig. 4D). Examination of propagation and activation maps for the different ChR2-CM groups (Fig. 4E) showed that ATR supplementation at 1 and 2 μM yielded smooth and fast propagation with similar CV to ChR2-CM control: 19.1 cm/s, 17.0 cm/s, and 17.0 cm/s for 0, 1, and 2 μM, respectively, while 4 μM resulted in small conduction disturbances (likely due to increased number of dead myocytes) and a drop in CV to 14.7 cm/s (Fig. 4F). In control CM, ATR had no effect on conduction velocity.

Overall, low concentrations of ATR (up to 2 μM) helped improve survival of cardiomyocytes in standard culturing conditions, did not alter conduction and resulted in significant APD prolongation but only slight prolongation of intracellular calcium transients in the ChR2-expressing cardiomyocytes.

## Discussion

### Dramatic optical energy benefits of ATR supplementation in cardiac cells

Means to manipulate cell electrophysiology optogenetically using low light are of great interest because of simpler setups (LED light sources vs. lasers) and avoidance of potential side effects on the cells, e.g. thermal effects caused by higher-intensity light (see Supplement in our previous publication^14^). So far, mutagenesis has produced a number of opsins with enhanced photocurrents and/or enhanced light sensitivity, including the recent super-sensitive ChR2-XXL, but in all cases, these optical enhancements come with a sacrifice in kinetics^29^. Such slow kinetics may not be ideal for pacing applications. Enhancement of optogenetic response by ATR or synthetic ATR mutants^30^, on the other hand, can theoretically yield low-light operation with less interference with opsin kinetics. When supplemented exogenously, as nutrients, Vitamin A derivatives can be transported via the vasculature into tissues^31^, and the photosensitizer ATR can be recruited to the cytoplasm to help form functional ChR2 complexes by covalently binding channelopsins^20^. Such diet-mediated delivery has worked in C. elegans^21^ and D. melanogaster^24^. As ATR is the chromophore for mammalian and microbial opsins, higher ATR concentrations are expected to increase light responsiveness, in general. Yet modulation of optogenetic responsiveness of mammalian cells by ATR has not been systematically studied before.

The baseline optical excitability of the cardiac syncytia, used in this study, without exogenous ATR, was excellent, i.e. using adenoviral delivery in cardiomyocytes we achieved irradiances needed to excite well under 1 mW/mm^2^ even for short pulses. Yet, the application of very low ATR doses (<2 μM) led to a further significant reduction in irradiance for excitation. While the photosensitizing effect of ATR on the ChR2 operation was expected based on prior studies in cells and organisms lacking endogenous retinal^21^,^24^, the surprising finding here is the extent of such enhancement in optical excitability of cardiomyocytes – 1–2 orders of magnitude across light pulse durations (Fig. 3). In contrast, in previous studies in neurons, ATR supplementation was not considered a factor in optical excitability^25^, presumably because of sufficient/saturating levels of ATR for ChR2 operation in neural cells, grown in B-27 supplemented medium, which contains some (undisclosed) amount of retinyl acetate. The implications of our findings are that when ATR is supplemented in low concentrations, extremely low light levels may be suitable for cardiac applications (this is the first study to report light levels needed to excite ChR2(H134R) down to a couple of μW/mm^2^), including optical pacing and cardioversion/defibrillation. This is important because such low threshold light levels are in the range of a variety of newer miniaturized and implantable low-power and/or remote-powered solutions for potential *in vivo* operation^32^,^33^, though such *in vivo* testing was not demonstrated here and is beyond the scope of this report. Further studies need to be conducted in adult cardiac tissue, which is denser, better coupled and presumably harder to excite, to see if controlled ATR supplements may help reduce the required light levels. Since all current optogenetic actuators of voltage (depolarizing and hyperpolarizing) use ATR as a chromophore, our findings have broader implications beyond the effects on ChR2 alone.

### From augmentation of ChR2 expression to improvement of optical excitability

Recently, Nagel and colleagues have elucidated the stabilizing role of ATR as a cofactor in the formation of functional ChR2 channels by showing that the absence of retinal results in faster degradation of ChR2^28^. Here in cardiomyocytes, we demonstrate that addition of very low concentrations of ATR increases ChR2 expression several-fold, as measured by the reporter eYFP (Fig. 2). This 5–6 times increase in ChR2 expression (likely through increase in the number of functional ChR2 channels in the membrane) then seems to result in much larger (over 30 times) augmentation of optical excitability for supplementation with 1–2 μM ATR (Fig. 3), as discussed above.

**Figure 5:**
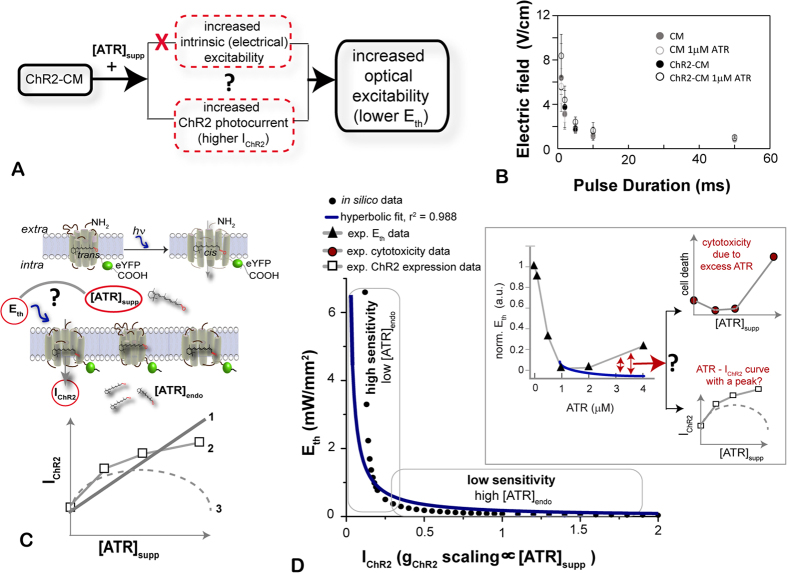
Theoretical explanation of optical excitability and sensitivity to supplemental ATR. (**A**) Two possible paths to the observed increase in optical excitability by the addition of ATR. (**B**) Electric field thresholds for excitation are not significantly altered by adding ATR, thereby eliminating the first possibility in (**A**), while indirect evidence exists for the second possibility (Fig. 2). (**C**) Light stimulation triggers retinal’s isomerization from *trans* to *cis* form, which in turn leads to ChR2 opening and net inward current. Several possibilities exist for the relationship between I_ChR2_ and [ATR]_suppl_, including simple linear scaling (1), saturating curve (2), as indirectly suggested by our ChR2 experimental data, or a hypothetical non-monotonic parabolic relationship with an optimal ATR concentration (3). (**D**) From assumed monotonic relationship between I_ChR2_ and [ATR]_suppl_ (we used the linear curve type 1), we calculate the optical *E*_*th*_ to stimulate human cardiomyocytes by light as function of [ATR]_suppl_ (*in silico* data); overlaid is a simple hyperbolic curve fit. Two distinct regions exist—of high sensitivity to ATR (if the endogenous ATR levels are low, possibly like found in cardiac tissue), and low sensitivity to ATR (for saturating ATR levels, possibly like neuronal or retinal tissue). Note that a saturating curve (type 2 in panel **C**) would yield a similar but steeper relationship. The inset replots our experimental data from Fig. 3, and red arrows indicate that the theoretical curve and the experimental data differ after ATR = 1 μM. This is likely due to cytotoxicity caused by excess ATR, independent of the ATR-ChR2 interactions, e.g. cell viability change as seen in Fig. 1 (replotted upper right panel) or, less likely, due to a non-monotonic parabolic relationship between I_ChR2_ and [ATR]_suppl_, which would mean that functional I_ChR2_ data does not follow the expression levels (see overlaid data from Fig. 2 in the lower right panel).

Theoretically, an increase in the ATR-mediated optical excitability in our experimental system can result in two ways (Fig. 5A): 1) exogenous ATR may act as a sensitizer of the cardiomyocytes, increasing their intrinsic excitability, independent of ChR2, and therefore reducing the opposing currents (to ChR2) and/or altering cell-cell coupling to trigger activity; and 2) exogenous ATR may augment the macroscopic ChR2 photocurrent, potentially by stabilizing more functional ChR2 molecules in the membrane at any given time, as suggested by the expression levels shown in Fig. 2. Using electrical stimulation, we tested the first possibility and found no evidence for supplemental ATR increasing the intrinsic excitability of the cardiac syncytium (Fig. 5B). Similarly, there was no evidence of increased self-oscillatory activity in the ATR-treated samples (CM or ChR2-CM), which would have been consistent with enhanced excitability. Therefore, indirectly, by elimination, we believe that the most plausible factor leading to increased optical excitability is indeed an ATR-augmented ChR2 photocurrent, likely by a previously proposed stabilizing mechanism for the ChR2 channels^28^. Even though our fluorescent data support such mechanism, a limitation of this study is that we did not directly measure the ChR2 photocurrents in cardiomyocytes under different ATR treatments.

Furthermore, we considered the relationship between the optical energy required for tissue excitation and the ChR2 photocurrent, I_ChR2_ (and its augmentation by ATR supplement), Fig. 5C,D, i.e. how does a five-fold increase in ChR2 expression translate into 1–2 orders of magnitude increase in optical excitability? Using a computational model of ChR2 in (human) cardiomyocytes, we have examined the relationship between irradiance needed to trigger an action potential and ChR2 conductance (see Supplement in our previous publication^14^). Current biophysical models of ChR2^14^,^22^ consider a simple scaling effect of ATR on ChR2 conductance (g_ChR2_), i.e. the g_ChR2_ can be alternatively viewed as ATR concentration (linear curve 1 in the bottom panel of Fig. 5C); a more biologically-realistic curve would be a non-linear saturating increase (curve 2 in Fig. 5C, which is also suggested by the ChR2 expression data from Fig. 2); a third possibility is a purely hypothetical non-monotonic parabolic curve (curve 3 in Fig. 5C), where an optimal ATR concentration may exist, beyond which excess ATR actually interferes with ChR2 function and reduces I_ChR2_.

Using the linear curve 1, we show the model-generated dependence of the threshold irradiance for cardiac excitation, *E*_*th*_, on supplemental ATR, Fig. 5D; note that using a nonlinear saturating curve 2 would yield a similar but steeper monotonically decreasing relationship. A hyperbolic curve is fit for the *E*_*th*_ as function of ATR – such theoretical (1/x) dependence is expected for an ideal rectangular current, where a lower ChR2 conductance (lower peak current) would require a corresponding increase of stimulus strength to maintain the same charge needed to excite. For the light- and voltage-dependent ChR2 current, such a fit is only qualitative because the provided current pulse is not rectangular, i.e. the ChR2 current waveform is feedback-regulated by the membrane voltage^12^. Two distinct regions of sensitivity of *E*_*th*_ to ChR2 conductance (or indirectly to ATR availability) are seen in Fig. 5D: 1) a high sensitivity region (for low endogenous ATR levels, as perhaps seen here for cardiomyocytes) and 2) a low sensitivity region (for saturating ATR levels, as perhaps seen in retinal and neural tissues). Operation in the steep-slope region (high sensitivity to ATR) can explain how a 5–6 times increase in ChR2 conductance may yield 30 times decrease in irradiance needed to excite cardiac cells, as reported here. Comparison of our experimental data for *E*_*th*_ (replotted example from Fig. 3 into the inset of Fig. 5D) to the theoretical predictions reveals similarities in the steep declining portion, but also differences – an increase in *E*_*th*_ beyond 1 μM ATR (red arrows). A likely explanation for this rise in *E*_*th*_ comes from the ATR-related cytotoxicity data (replotted from Fig. 1). Akin to receptor-mediated hormonal action^34^, ATR seems to operate within a well-defined optimal range in cardiac cells (see below), and a decrease in cell viability is seen >2 μM ATR, which can explain the reduced optical excitability. Such cytotoxic effects are not related to ChR2-ATR interactions per se and are not part of the model predictions. An alternative, less likely, cause for the deviation between experimental and theoretical data is the possibility for a non-monotonic parabolic relationship between supplemental ATR and I_ChR2_ (hypothetical curve 3 in Fig. 5C), which would be in contrast to the expression data from Fig. 2, showing total channelopsin expression with or without bound ATR. A more mechanistic study to test such proposition would require extensive photocurrent characterization under different conditions.

### ATR effects on cardiac electrophysiology: narrow range of safe ATR supplementation in cardiac tissue

This study presents a first glimpse at the electrophysiological effects of optogenetically modifying cardiomyocytes (directly by adenoviruses) to express the light-sensitive actuator ChR2. Similar to our earlier findings using dedicated ChR2-expressing donor cells coupled to non-modified CMs, employing the “tandem-cell-unit”^15^, we find here no significant alterations in the action potentials, calcium transients or conduction velocity between control CMs and genetically modified ChR2-CMs (Fig. 4). This is an important baseline characterization in order to use optical actuation as a “clean” tool for affecting cardiac function.

Furthermore, in this study, we quantified the effects of supplemental ATR on cardiomyocyte electrophysiology. For low doses of supplemented ATR (<4 μM), the ChR2-CM exhibited significant prolongation of APD80 (44%) and small CTD80 prolongation (<15%), without significant effects on conduction velocity, Fig 4. The APD prolongation was only seen in ChR2-CMs and not in the control CM samples supplemented with 1 μM ATR, which points to ChR2-dependent effects of ATR on repolarization. One possibility is that the enhanced light sensitivity by ATR supplementation affects the entire spectrum of ChR2 excitation, bringing about some unexpected responsiveness to the longer wavelength (660 nm) used for voltage dye imaging, at which normally ChR2 should not have any absorption. If so, some small residual depolarizing current via ChR2 may indeed prolong the APD. However, further studies are needed to fully understand the mechanism of these electrophysiological changes and the potential ion currents involved.

Overall, our results indicate high sensitivity of cardiomyomyocytes to exogenous ATR – in this *in vitro* system, the optimal range was determined to be 1–2 μM, with a sharp increase in toxicity above 4 μM (Figs 1 and 4), regardless whether the cells expressed an opsin or not, but this ATR-mediated cytotoxicity seems to be enhanced for ChR2-CM compared to CM (Fig. 1). This is not surprising considering the critical role retinoids play as potent regulators of gene expression and cell fate. Believed to control over 500 genes^35^, retinoids play a major role in cell growth, differentiation in embryogenesis, and in the adult stage. Among other effects, they also have been shown to augment differentiation of embryonic stem cells into ventricular cardiomyocytes^36^; in zebra fish, immediately after ventricular injury, an upregulation in the cardiac production of retinoic acid was found, essential for heart regeneration^37^. The concentrations of intermediary products in the retinoid metabolism are likely tightly regulated to avoid undesired effects. For example, excess exogenous ATR is likely converted to retinoic acid (RA) and can result in elevated RA levels. Endogenous RA levels are maintained in a narrow range (1–4 nM) and excess RA is cytotoxic^38^, known to engage apoptosis-related signaling pathways. Therefore, excess RA is a likely toxicity driver in our results with ATR concentrations higher than 4 μM (Fig. 1). Alternatively, excess ATR itself (not used by the opsins and not converted to RA) may be toxic. ATR has been reported as a competitive blocker of cyclic-nucleotide-gated (CNG) ion channels^39^. Previously characterized are cGMP-gated (rod) ion channels, showing full inhibition at 4 μM ATR, coincidentally, on par with concentrations at which we detected cytotoxicity here. One can speculate that, at sufficiently high doses, ATR action may extend to other ion channels relevant to cardiac electrophysiology (cyclic-nucleotide-gated, e.g. HCN, or not). Either of these can explain the ATR’s impact on cardiac electrophysiology and its clear toxicity outside of the narrow range of 1–2 μM, found here. A limitation of this study is that only *in vitro* testing was conducted using multicellular cardiac samples prepared with neonatal rat cells, which have different electrophysiological characteristics than adult cardiomyocytes. Future studies in intact adult animals are needed to confirm our predictions (obtained *in vitro*, in neonatal myocytes) for ATR’s potent light-sensitizing effects at small doses to lower the optical energies needed in cardiac applications of optogenetics. We conclude that cardiomyocytes, as tested in this *in vitro* study, seem to operate at non-saturating retinal levels, and carefully-dosed exogenous ATR can enhance the performance of ChR2 in cardiac cells and yield orders of magnitude energy benefits for optogenetic stimulation.

## Materials and Methods

### Primary cardiomyocyte cell culture

Primary cardiomyocytes (CM) were isolated as reported previously^15^,^40^,^41^. Briefly, ventricular tissue was harvested from 2 to 3 day old Sprague-Dawley rats, and digested with 1 mg/ml trypsin (US Biochemicals, Cleveland, OH) in Hanks’ balanced salt solution (HBSS, GIBCO Invitrogen, Carlsbad, CA) at 4 °C overnight, followed by serial digestion using 1 mg/ml collagenase (Worthington Biomedical, Lakewood, NJ) in HBSS. After centrifugation, the cell pellets were re-suspended in culture medium M199 (GIBCO) supplemented with 12 μM L-glutamine (GIBCO), 0.05 μg/ml penicillin-streptomycin (Mediatech Cellgro, Kansas City, MO), 0.2 μg/ml vitamin B12 (Sigma, St. Louis, MO), 10 mM HEPES (GIBCO), 3.5 mg/ml D-(+)-glucose (Sigma) and 10% fetal bovine serum, FBS (GIBCO). CMs were separated from fibroblasts by two 45 minute pre-plating steps, and counted before further processing.

### Adenoviral ChR2 expression in cardiomyocyte syncytia

Adenoviral delivery of ChR2(H134R) in cardiomyocytes was done by infection in suspension, as described previously^11^. Briefly, using plasmid pcDNA3.1/hChR2(H134R)-EYFP from Addgene (Cambridge, MA), we developed an adenoviral construct (pBR322 backbone) with a ubiquitous CMV promoter. First-generation adenovirus was generated by homologous recombination of the Ad-CMV-ChR2-eYFP into pTG3604; further propagation and purification of the virus genomes was done by transfection into HEK293 cells and CsC1 banding. Purified CMs were incubated in Ad-ChR2-eYFP-containing M199 medium (or just M199 for controls) with 2% FBS for 2 hours with gentle agitation every 20 min in humidified environment. Optimized multiplicity of infections (MOI) of 25 (and 15) demonstrated over 98% expression using a 10^11^ units/ml (and 10^12^ units/ml) titer, respectively for two batches of the virus, without inducing any increased cell death compared to control, as described previously^11^. After incubation, virus was promptly removed and replaced with culture medium with 10% FBS, supplemented with ATR (0 to 10 μM), as desired.

ChR2-CM were plated on fibronectin-treated glass bottom dishes (14 mm in diameter) at a density of 400 k/cm^2^ to form well-connected CM monolayers (syncytia), and incubated in 5% CO_2_ at 37 °C. On day 1, all samples were washed with PBS for 5 minutes with gentle shaking in dark and continued to be incubated in ATR-supplemented media with 10% FBS until day 3, when the ATR culture medium was switched to 2% FBS and exchanged every other day. All functional electrophysiological experiments were conducted on day 4 with Tyrode’s perfusion maintained at 30 °C throughout data acquisition, as done before^15^. Samples used for imaging (of cell viability or ChR2 expression) were also utilized on the same day.

### All-trans-Retinal (ATR) treatment

All-*trans*-Retinal (Sigma) was first dissolved in filtered DMSO (Sigma) to a stock concentration of 100 mM, and further diluted in M199 growth medium (GIBCO) with 10% or 2% FBS to final concentration of 1 to 10 μM. Prior literature shows that concentrations of <0.02% DMSO do not have undesirable effects on neurons nor cardiomyocytes^42^,^43^. The growth media supplemented with ATR was filtered again before use. CMs were grown in ATR-containing medium with replenishment as described, up to the time of viability assay and experimentation. The experimental groups included both control and Ad-ChR2(H134R)-eYFP-infected samples, treated with the selected ATR concentration (0, 0.1, 0.5, 1, 2, 4, 8, and 10 μM), i.e. 16 groups total.

### Quantification of cell viability and ChR2 expression by image analysis

Images were acquired using the Olympus FluoView^TM^ FV1000 confocal system, maintaining similar gain and exposure values across conditions for further processing. Image analysis and quantification were done using custom-developed software in Matlab (Mathworks, Natick, MA).

Cell viability was assessed by detecting dead cells’ DNA fragments by staining with 2 μg/ml Propidium Iodide (PI) (GIBCO) in Tyrode’s solution on the 4^th^ day after isolation and infection. Quantification of cell death was done by removing image background based on a constant threshold for all groups and summing up the PI fluorescence pixels. Summed PI pixels of images of CM control with no ATR (18 FOV) were then averaged, and the mean served as a reference of normalization for other conditions. The summed PI pixels of images from all groups were then normalized to CM control PI average to calculate fold change.

ChR2 expression was judged by eYFP fluorescence, after the samples were fixed in 3.7% formaldehyde on the 4^th^ day after infection, followed by permeablization in 0.02% Triton X-100 and nuclei stain with 1 μg/ml 4′,6-diamidino-2-phenylindole (DAPI). ChR2 expression was quantified by eYFP fluorescence as follows: Images of control CMs treated with different concentrations of ATR were processed to determine a noise threshold (for each ATR treatment), below which all green pixels were considered to be caused by autofluorescence. These noise thresholds were then used (by subtraction) to correct for background in ChR2-CM images of the corresponding ATR concentration. Thus corrected eYFP readouts were normalized by cell number from DAPI staining in each image. Finally, to present the data as fold-changes, results were pooled from multiple cultures, and the corrected and cell-normalized values for each ChR2-CM group, treated by ATR, were further divided by the mean value from the zero ATR ChR2-CM group.

### Electrophysiological characterization: electrical stimulation, optical actuation and optical mapping

In order to characterize wave propagation in the CM monolayers, electrical stimulation (pulses 10 V, 0.005 s, 1 Hz) was delivered through a bipolar platinum electrode connected to a pulse generator (IonOptix, Milton, MA). Electric field stimulation (with a pair of line electrodes, spaced 8 mm apart) was used for testing the electrical excitability (variable field strength and pulse duration, 1 Hz pacing). Optical stimulation (470 nm, variable pulse width, 1 Hz) was applied as global illumination of the sample from underneath (area of illumination about 10 mm in diameter), using a collimated beam generated by an optical fiber-coupled laser (Shanghai Laser, Shanghai, China) driven by a TTL pulse generator (IonOptix). Optical excitation thresholds were determined as the minimum optical energy needed to induce ChR2-CM activation at different pulse durations. Optical energy (mW/mm^2^) was measured by a digital power meter (Thorlab, Newton, New Jersey) with a sensor (diameter 9.5 mm) behind the same type of glass bottom dish.

Optical imaging of voltage or calcium was performed using optical probes, spectrally compatible with ChR2. Specifically, action potential measurements were done with the red-shifted voltage-sensitive dye Di-4-ANBDQBS (35 μM, from L. Loew, University of Connecticut), excited with a red LED (660 nm, Thorlabs), emission >700 nm. Intracellular calcium was imaged with Rhod-4AM (10 μM, AAT Bioquest, Sunnyvale, CA) with fluorescence excitation at 530 nm (green LED, Thorlabs) and emission at 605 nm. Macroscopic wave propagation was imaged as described previously^15^,^44^ using a custom-built system with a Navitar Platinum lens (50 mm, f/1.0) and an intensified-CMOS camera, 1024 × 1080 pix (pco, Germany), binned 2x, at 200 fps full frame. An EMCCD camera (iXon Ultra 897 EMCCD; Andor, UK), run at 300 fps, was used for voltage measurements.

Data was acquired continuously using CamWare interface (pco) or NIS-Elements AR (Nikon Instruments; Melville, NY), and analyzed using custom-developed software in Matlab that automatically detects events (action potentials or calcium transients) and characteristic parameters associated with these; furthermore, it generates activation maps and Hilbert-phase movies of propagation^44^. Data pre-processing included baseline removal, contrast improvement by scaling to full dynamic range, a Bartlett spatial filter (3 × 3 window) and a temporal Savitzky-Golay filter (second order, 7 frames window). Action potential duration and calcium transient duration at 80% (APD80 and CTD80) were determined as the time difference between the beginning of a transient and the point of 80% recovery from the peak. Each sample’s APD80 and CTD80 are an average of 3–4 beats. Activation maps were generated based on automatic detection of the time of maximum rise. Isochrones were marked by the frames with 10 ms difference. Conduction velocity (CV) was calculated by sampling points along straight paths in the activation maps (distance traveled divided by the timing between frames).

### Statistics

Image analysis of ChR2 expression, PI, and other functional. Threshold irradiance data on strength duration curves were analyzed using 2-way ANOVA.

## Additional Information

**How to cite this article**: Yu, J. *et al.* Cardiac Optogenetics: Enhancement by All-*trans*-Retinal. *Sci. Rep.* **5**, 16542; doi: 10.1038/srep16542 (2015).

## Supplementary Material

Supplementary Information
