# Supplementary material for: Cardiac Optogenetics: Enhancement by All-trans-Retinal
Source: Sci Rep. 2015 Nov 16;5:16542. doi: 10.1038/srep16542 (PMC4644984; doi:10.1038/srep16542)
Supplement: Supplementary Information [file srep16542-s1.pdf]

Cardiac Optogenetics: Enhancement by All-Trans-Retinal

Jinzhu Yu, BS[#], Kay Chen, BS[#], Rachel V. Lucero, Christina M. Ambrosi, PhD, and Emilia Entcheva, PhD*

Department of Biomedical Engineering, Stony Brook University, Stony Brook, NY

Supplemental Information for Yu et al.

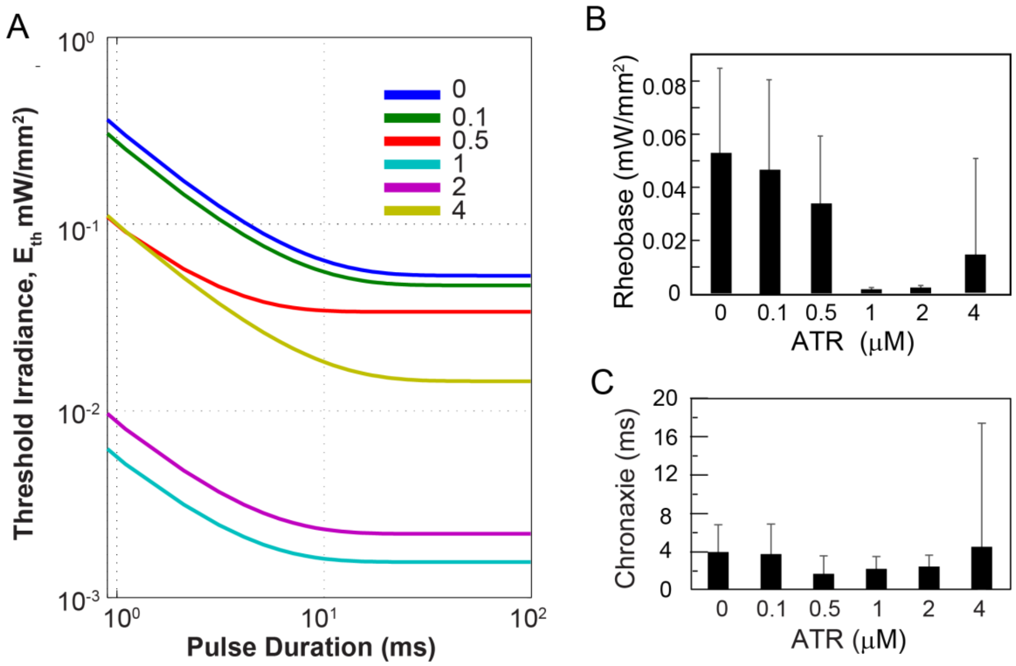

Supplemental Figure S1. Parameter fits to the strength-duration data in Fig 3. (A) Using the Matlab curve fitting tool box, fitting to the model $\frac{Rheobase}{1-e^{\frac{-x\log(2)}{chronaxie}}}$ was completed within 4×10^6 iterations and termination tolerance = 10^{11}. (B) Fitted rheobase with 95% confidence intervals (CI). The CIs of 1 and 2 μM groups do not have any overlap with that of other concentrations. (C) Fitted chronaxie with 95% confidence intervals.

Supplemental Table S1. Outcome of statistical analysis of data from Fig 3 – significant differences indicated. The two factors are pulse duration and ATR concentration. (*) denotes a significant difference, detected in pair-wise comparisons after Tukey-Kramer post hoc correction and by non-overlapping confidence intervals with α level set at 5%. Since all pulse duration comparisons share the same pattern of significance (except 1 ms), comparisons among ATR concentration are listed in the table. The 1ms group did not achieve significant difference from the 0.5 μM condition.

	0	0.1	0.5	1	2	4
0				*	*	*
0.1				*	*	
0.5				*	*	
1	*	*	*			*
2	*	*	*			*
4	*			*	*	
